# Structural parameters optimization for enhanced sealing performance of soluble ball seat sealing ring in shale gas volume fracturing process

**DOI:** 10.1371/journal.pone.0332824

**Published:** 2025-10-09

**Authors:** Shuang Jing, Anle Mu, Zhen Chen, Qingjie Ran, Deyong Luo, Tao Xiong

**Affiliations:** 1 School of Mechanical and Precision Instrument Engineering, Xi’an University of Technology, Xi’an, Shaanxi, China; 2 MOE Key Lab of Oil and Natural Gas Equipment, Southwest Petroleum University, Chengdu, Sichuan, China; 3 Petroleum and natural gas equipment technology, Sichuan Province science and technology resource sharing service platform, Chengdu, China; 4 Petrochina Southwest Oil and Gas Field Company Sichuan Northwest Gas Mine, Chengdu, China; University of Oxford, UNITED KINGDOM OF GREAT BRITAIN AND NORTHERN IRELAND

## Abstract

In the volumetric fracturing process of tight oil and gas horizontal wells, the soluble ball seat emerges as a crucial tool for pressure sealing due to its rapid solubility, full-diameter production capacity, and straightforward construction process. This study focuses on enhancing the sealing efficacy of metal sealing rings paired with soluble ball seats. Employing finite element analysis, structural optimization, and indoor diameter reduction tests, we assess the sealing performance of metal sealing rings fabricated from traditional Al-Mg alloy (Material 1) and a modified Al-Mg/Ga alloy (Material 2). By establishing a comprehensive evaluation system for the sealing performance and utilizing finite element simulation, we compare the mechanical properties and sealing effectiveness of both materials during the setting process. Our findings reveal that Material 1’s sealing ring endures stresses surpassing allowable limits, heightening the risk of local failure. Conversely, structural optimization of the Material 2 sealing ring allows for safe installation under a standardized 5T setting force, ensuring that contact stress between the sealing ring, casing inner wall, and the sliding body exceed the fracturing fluid pressure with a relatively uniform stress distribution. Subsequent indoor diameter reduction tests verify the superior safety performance of Material 2 over Material 1, aligning with the finite element analysis outcomes. Collectively, this research delineates how finite element analysis, structural optimization, and empirical tests augment the structural safety and sealing functionality of metal sealing rings for soluble ball seats, furnishing a basis for refining the design of soluble ball seat sealing rings.

## 1. Introduction

As China intensifies its pursuit of energy self-sufficiency amid rising dependence on foreign oil and gas resources, enhancing domestic exploration and production has become critically important. With conventional reserves proving insufficient to meet growing demand, unconventional resources such as tight oil and gas have become central to the nation’s energy strategy [1–3]. The poor physical properties of these unconventional reservoirs, combined with significant heterogeneity from natural fractures, render traditional hydraulic fracturing economically inefficient and technically inadequate [4–6]. Volumetric fracturing technology has emerged as a promising solution, transforming the efficient development of tight reservoirs and substantially increasing production yields. Key to this technology’s success is the rapidly dissolvable ball seat, widely adopted in volumetric fracturing operations due to its rapid dissolution, full-bore production capability, and simple installation process. This innovation has significantly advanced multi-cluster horizontal well fracturing techniques [7–8].

Extensive literature examines the structural integrity and sealing capabilities of bridge plug technologies [9–15]. In China, Yue adopted a predictive approach to appraise the sealing comportment of bridge plug rubber cylinders, harnessing a combination of BP network analyses and orthogonal testing methods, with the analytical robustness of the ABAQUS software platform as the proving ground for the feasibility of the predictive methodological framework. Liu conducted rigorous laboratory and field experiments on soluble bridge plugs in horizontal shale gas wells, critically evaluating their adaptiveness to field-level application conditions and confirming the stability of bridge plug’s pressure-sealing comportment and its impressive solubility post-fracturing operations. Guo’s research focused on the structural design and application of high-strength soluble bridge plugs, supported by laboratory and field assessments, which highlighted their stable sealing performance and reliable setting. International researchers, including Qu, have made strides by constructing finite element models for dual-cylinder rubber configurations and scrutinized the impact of friction coefficients pertaining to sealing elements on the holistic sealing system’s energetic efficiency through orthogonal test methods. These studies underscored that attenuation of the frictional interactions between the casing and the rubber cylinder is pivotal to averting potential rubber cylinder impairments. Gang and his colleagues pursued the influence exerted by rubber cartridge material selections on compression packers’ sealing efficacy under disparate setting forces employing finite element methodologies, culminating in the insight that judicious material selections could conspicuously amplify sealing performances. Lan and collaborators leveraged the ABAQUS suite to facilitate a numerical analysis and structural optimization of rubber cylinders crafted from diverse materials, culminating in an innovative single-cylinder design augmented with an expansion support ring, validated for its superiority. Bi-metal seal is also carried out related research both at home and abroad, Kim’s inquiries into conventional O-ring metal seal designs tackled issues associated with inadequate metal springback capabilities, leading to topological optimization of the O-ring’s shape. Successively, based on these optimized outcomes, Kim undertook a structural optimization design of the O-ring metal, garnering a version with heightened springback potency. Considering the squeeze-induced contact between sealing surfaces, the surface roughness is a substantial determinant of their sealing potential. Wang ventured into finite element analyses of metallic sealing rings utilized in subsea pipeline connectors, characterizing their sealing acumen through their stress states, compressive springback measures, and contact stresses, which collectively serve as harbingers of the material’s damage resilience, springback characteristics, and sealing reliability. This nexus was further correlated with the initial compression proportions to deduce the practicability of the prestressing installation procedures. Yang formulated a theoretical exposition for the contact stress dynamics characteristic of metal seals in intelligent well downflow control valves, corroborated through numerical analyses using the ABAQUS software ensemble [16–20].

However, research on the structural and sealing performance of soluble ball seats for fracturing remains limited. Current evaluation methods primarily rely on empirical field tests or simulations, lacking standardized benchmarks for metal sealing performance. This study therefore addresses the critical issue of metal sealing ring failure during setting and fracturing operations. By establishing a sealing performance evaluation model for soluble ball seat metal sealing rings and conducting finite element simulations under various setting and fracturing conditions, we systematically investigate their structural integrity and sealing behavior. Material improvements and structural optimization significantly enhance both sealing effectiveness and safety of these metallic components, enabling compliance with demanding horizontal well fracturing requirements. The evaluation and optimization methodologies proposed herein provide foundations for advancing metal sealing technologies.

## 2. Analysis of the setting and sealing mechanisms of the soluble ball seat sealing ring

**Fig 1:**
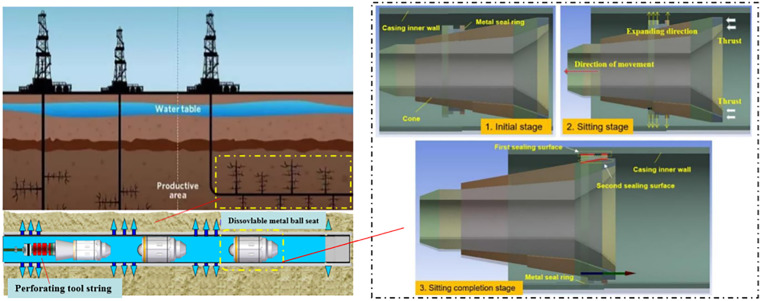
Schematic diagram of soluble ball seat sealing ring working principle.

The working principle of the soluble ball seat sealing ring, as depicted in Fig.1, involves a detailed process initiating with the application of a setting force. During the activation of the soluble ball seat, the sliding body exerts a compressive force upon the sealing ring. This force instigates a radial extrusion, compelling the sealing ring to expand outwardly. The consequent expansion facilitates the outer region of the sealing ring to make contact with the inner wall of the casing, thus achieving the seal’s setting phase. Concurrently, the outer periphery of the sealing ring undergoes compression by the casing wall. The efficacy of the seal during fracturing is contingent upon the resultant contact stress between the outer side of the sealing ring and the casing’s inner wall, in addition to the generated contact pressure between the sealing ring’s inner wall and the sliding body. This delineates two pivotal sealing interfaces: (1) the interactional surface between the exterior of the sealing ring and the casing’s inner wall; (2) the interface promoting sealing efficacy between the internal wall of the sealing ring and the sliding body. Significantly, the integrity of the seal between the outer sealing ring and the casing’s inner wall is instrumental in ascertaining the overall sealing performance of the ball seat.

## 3. Establishment of sealing performance evaluation metrics for the metal sealing ring of soluble ball seat

In volumetric fracturing for tight oil and gas development, the sealing performance of soluble ball seats critically impacts operational success. While seal effectiveness is typically evaluated empirically, simulation-based assessment requires specialized metrics due to the difficulty of directly measuring sealing capacity. This study addresses this gap by establishing comprehensive evaluation criteria for soluble metal sealing rings, adapting and extending existing bridge plug rubber seal standards. We introduce a multi-metric approach incorporating material properties, equivalent stress, and contact stress characteristics (maximum, average, and variance) as key performance indicators. This integrated evaluation framework advances sealing performance assessment methodologies, establishing new benchmarks for industrial and academic applications [21–26].

### 3.1. Structural strength criteria

In the setting process of the metal sealing ring within the ball seat, it is crucial to ensure the ring’s integrity. Therefore, the maximum equivalent stress endured by the sealing ring must not exceed the material’s allowable stress threshold. This can be mathematically represented as:


$$\sigma <[\sigma_{\text{b}}]$$ (1)

Where, σ is the equivalent stress of metal seal ring during setting; [$\sigma_{\text{b}}$] is the permissible stress of metal sealing ring material.

### 3.2. Structural strength criteria

Evaluating the contact pressure between sealing components is typically utilized as a standard metric for assessing sealing efficacy. An optimal seal is characterized by higher contact pressures along the sealing interface, which should ideally be as uniform as possible to avoid seal failure. Guided by the sealing principles of metal rings, the principal metrics for assessing the sealing ring structure include (1) the maximum contact stress criterion and (2) the contact uniformity criterion.

(1) Maximum Contact Stress Criterion

During sealing, the maximum contact pressure exerted between the sealing ring and the casing must surpass the maximum anticipated hydraulic pressure to prevent any potential cross-flow of oil and gas.


$$P\geq P_{down}$$ (2)

Here, *P* alludes to the contact stress between the metal sealing ring and casing’s inner wall, while *P*_*down*_ represents the operating pressure differential for the soluble ball seat, typically in the magnitude of 50 MPa.

(2) Contact Uniformity Criterion

The uniformity of contact stress distribution across the metal ring directly corresponds to the seal’s performance. A severely uneven distribution can lead to seal malfunction, adversely impacting the formation of a stable metal seal interface. The performance indices for contact uniformity include the mean contact pressure and the variance of contact stress distribution on the sealing surface. To evaluate this, *N* equidistant points are selected on the sealing surface to compute the variance as follows:


$$\begin{array}{c} \overset{―}{p}=\frac{\text{1}}{n}\sum {\mathrm{\backslash nolimits}}_{i}^{n}p_{i} \end{array}$$ (3)

$$S^{\text{2}}=\frac{\sum_{i}^{n}(p_{i}-\overset{―}{p}{)}^{\text{2}}}{n-\text{1}}$$ (4)

Where $\overset{―}{p}$ is the average contact stress between the metal seal ring and the casing inner wall, $S^{\text{2}}$ is the variance of the contact stress on the sealing surface, and $P_{i}$ is the contact stress at the *i* th measured point.

## 4. Simulation analysis of the sealing performance of the metal sealing ring in a soluble ball seat

### 4.1. Creation of the finite element model for the soluble ball seat seal

**Fig 2:**
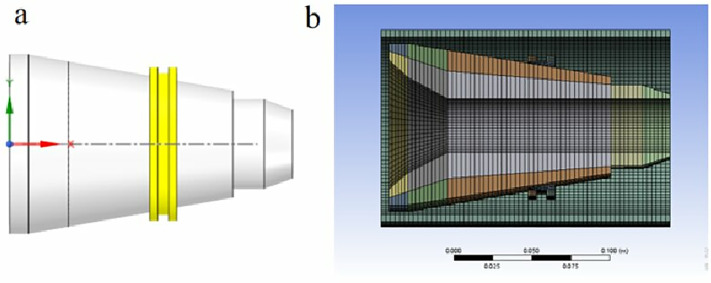
Simplified finite element model and mesh generation. **(a)** Simplified geometric model diagram. **(b)** Grids division of the simplified model.

This study simplifies the finite element model according to the soluble ball seat’s structure and working principle. To focus on how sealing ring dimensions affect sealing performance and safety, non-essential components (e.g., slips) were excluded, retaining only the sliding body, sealing ring, and casing. We used ANSYS Meshing to generate structured grids, applying quadrilateral face mapping to all cylindrical surfaces followed by swept meshing to create structured hexahedral elements with a 3 mm grid size. Fig 2 shows the simplified geometric model and mesh configuration, resulting in 48,482 elements and 70,349 nodes.

The soluble ball seat sealing ring is fabricated from two distinct materials:

(1) Material 1: A superplastic Al-Mg alloy
(2) Material 2: A modified Al-Mg alloy incorporating specific proportions of Ga, In, Zn, and Sn

**Fig 3:**
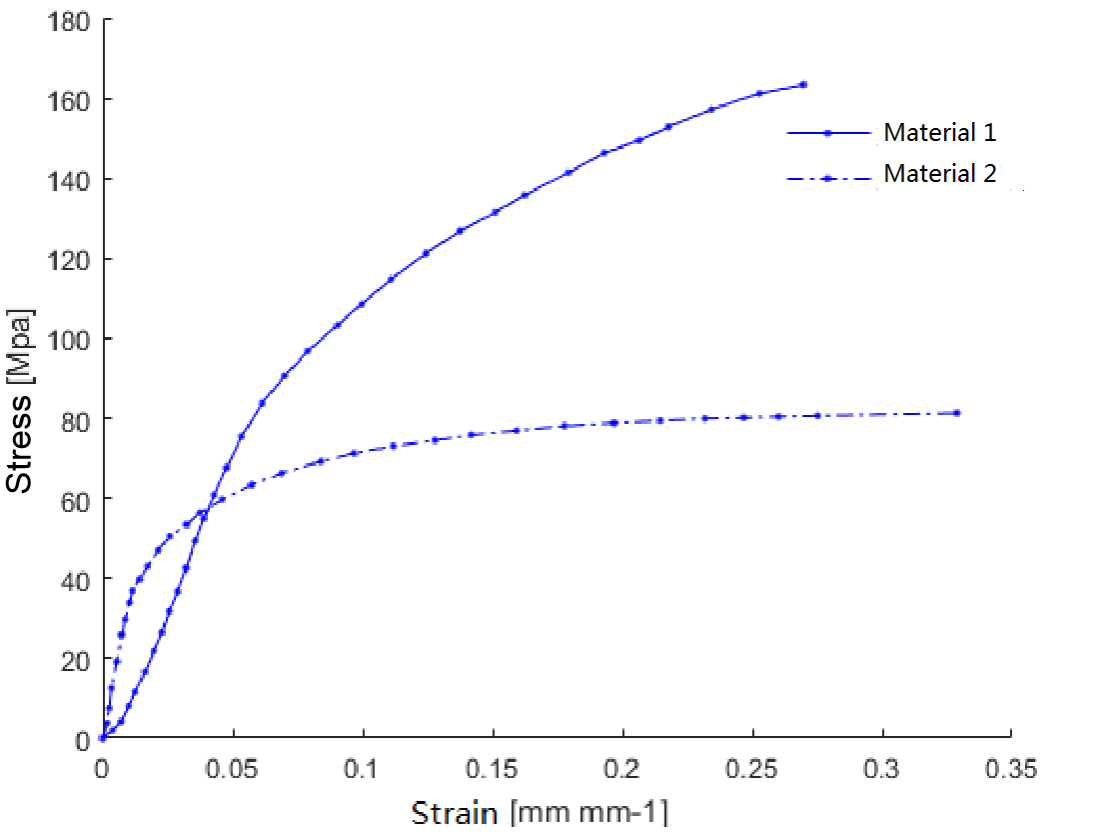
Stress-strain curves of both materials.

All other components utilize structural steel with default material parameters. The stress-strain relationships for both alloys are presented in Fig 3.

The stress-strain values for the materials in question were entered into ANSYS Workbench, where the Mooney-Rivlin 2-parameter model was employed to establish a curve fit. The resulting model parameters are detailed in Table 1. For the scenarios considered in this study, when the material of the sealing ring is designated as ‘Material 1’, the system operates under ‘Condition 1’; conversely, with ‘Material 2’ as the sealing ring material, the system functions under ‘Condition 2’.

**Table 1:** Parameters of Mooney-Rivlin model.

Material	C10 (Pa)	C01 (Pa)	D1 (Pa^-1)
Material 1	2.929E + 07	2.793E + 06	1.443E-07
Material 2	−7.498E + 06	2.559E + 07	1.443E-07

The sliding body is displaced rightward at its left end. A fixed support is applied to the casing exterior, while a 0 mm axial constraint is imposed on the sealing ring’s right face. The friction coefficient between sealing ring and casing wall is set to 0.3. Analysis settings employ automatic time-stepping with solution control adjusted to account for large deformations.

By enforcing the aforementioned boundary conditions, it becomes feasible to ascertain the reactive force on the sliding body’s left face for varying displacements, thereby deducing the consequent displacement of the sliding mechanism along with deformation, stress, strain, and contact pressure inflicted upon the sealing parts due to the application of the prescribed setting force (with a maximum value, Fmax, set at 5t).

### 4.2. Results analysis

**Fig 4:**
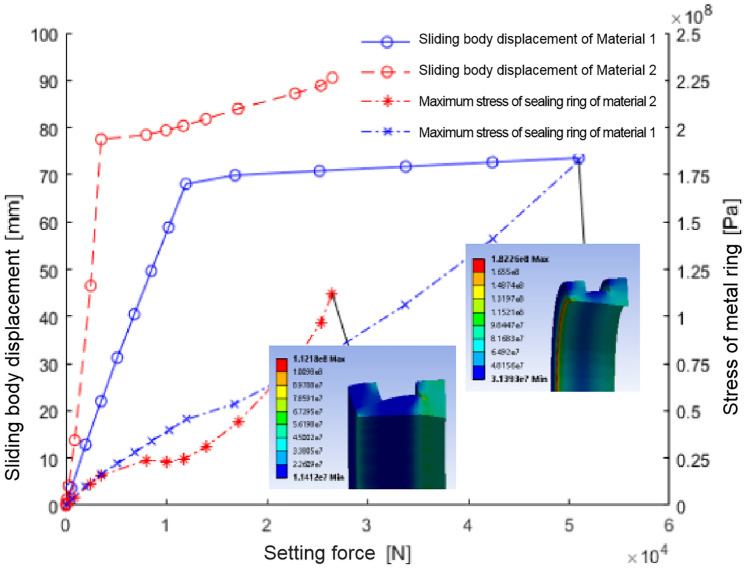
The relation diagram of sliding body displacement, maximum stress of metal ring and setting force during setting of sealing ring of two materials.

After implementing these parameters, the simulation provided insights into the metal sealing ring’s mechanical and sealing performance. Finite element analysis generated curves showing the relationship between sliding body position, maximum stress, and sealing ring setting force (Fig 4).

The sealing ring made of Material 1 shows a significant change in setting force at approximately 69 mm sliding body position

**Fig 5:**
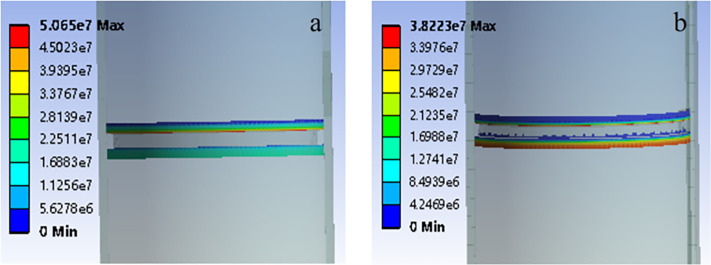
Contact pressure distribution between sealing ring and casing wall of two materials. (a) Material 1. (b) Material 2.

At this juncture, the sealing ring disengages and initiates contact with the casing wall (Fig 5). The sliding body’s continued movement furthers the setting process, and the setting force escalates. Upon reaching a setting force of 5T, the setting operation culminates. The sealing ring endures a stress of about 180Mpa, surpassing its permissible stress limit of approximately 161Mpa. The area of utmost stress concentration is the interface between the sealing ring and the larger diameter segment of the sliding body, rendering it susceptible to potential damage.

For ‘Material 2’, the sealing activation commences when the sliding position approaches 78 mm, making initial contact with the casing wall. As the displacement extends to 90 mm, the setting force registers approximately 2.65t. The stress on the sealing ring at this point is 112.18Mpa, which exceeds its designated allowable stress threshold of about 83Mpa. The stress peaks within the central groove of the sealing ring, signaling a probable site of impairment.

**Fig 6:**
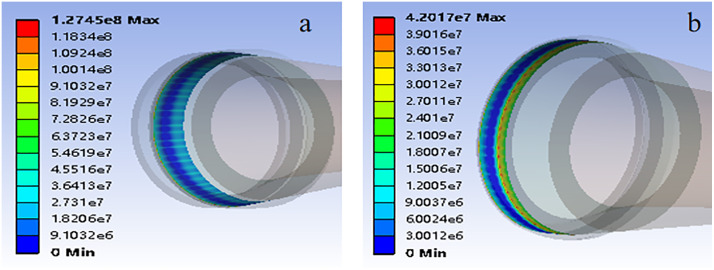
Contact pressure distribution between sealing ring and sliding body of two materials. (a) Material 1. (b) Material 2.

The seal integrity of the sealing ring fundamentally relies on the interface with the inner wall of the sleeve and the sliding body. To adequately assess the sealing efficacy, it is imperative to examine the contact pressure between the sealing ring, the sleeve’s inner wall, and the sliding entity. For both materials, the investigation yields the maximum and average contact pressures against both the casing wall (Pmax1, Pmean1, and σ1) and the sliding body (Pmax2, Pmean2, and σ2), as well as the variability in contact pressure (σ1 and σ2). These findings, alongside the contact pressure distribution, are graphically represented in Fig 6.

Analysis of Fig 6 and the compiled data in Table 2 reveals that when the sealing ring is fabricated from ‘Material 2’, the peak contact pressure exerted on both the casing wall and sliding body remains below 50 MPa. Furthermore, the contact pressure distribution along the interface demonstrates significant heterogeneity, characterized by a comparatively low mean contact pressure. A critical finding is the evident gap formation between the upper section of the sealing ring and the casing wall, accompanied by a loss of contact in the central region between the sealing ring and sliding body. Under the current structural configuration, the metallic sealing ring fabricated from Material 2 fails to maintain effective sealing performance when subjected to a differential pressure of 50 MPa.

**Table 2:** Comparative analysis table of performance of sealing ring of two materials.

Material	P_max1_(Mpa)	P_mean1_(Mpa)	σ1	P_max2_(Mpa)	P_mean2_(Mpa)	σ2
Material 1	50.6	16.82	94.55	127.45	20.16	422.28
Material 2	38.223	15	205.86	42	14.53	146.631

## 5. Material selection and structural optimization of the metal sealing ring for soluble ball seat

### 5.1. Material selection criteria for metal sealing rings

Comparing diameter expansion, peak contact pressure, and average contact force shows the original material generates slightly higher contact pressures against both sliding body and casing wall. However, it requires excessive setting force (5T) that causes over-expansion and exceeds material stress limits. While increasing force boosts contact pressure, it simultaneously amplifies seal stress. In contrast, the modified material’s sealing ring achieves higher contact pressure at lower setting force through structural optimization while maintaining safe stress levels, establishing it as the superior choice.

### 5.2. Structural advancements in metal sealing rings

The incumbent seal design presents issues such as uneven sealing, interspace on the contact surface, and excessive stress concentration, specifically within the groove region. These challenges are surmountable by increasing seal thickness adjacent to the actuation body. We introduce an innovative seal design and, using simulation, dissect the impacts of crucial structural factors—seal thickness (D1), groove profundity (D2), upper (θ1) and lower (θ2) groove angles, and contact angles with the slider (θ3) and casing wall (θ4)—on the sealing integrity and material endurance.

**Fig 7:**
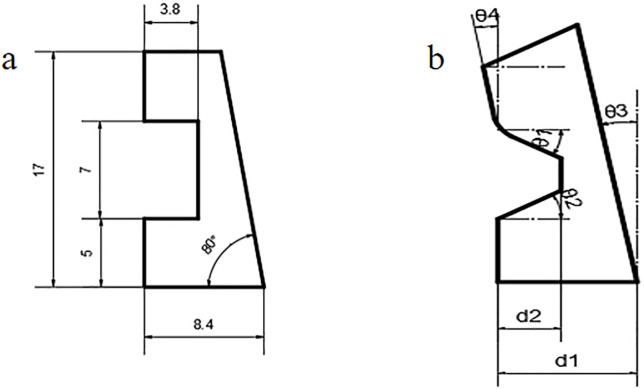
Comparison diagram between the improved structure and the original structure. (a) Original seal ring profile structure. (b) Improved seal ring profile structure.

Refer to Fig 7 for a comparative illustration of legacy and optimized seal profiles. Fig 7 depicts contrasted images of the (a) initial seal and (b) refined seal structures.

#### 5.2.1. Impact of seal thickness (D1).

**Fig 8:**
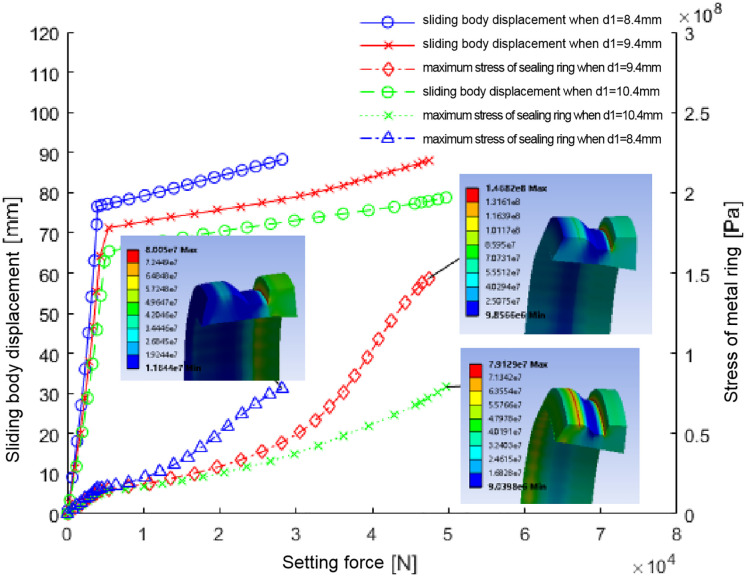
Diagram of relationship between sliding body displacement, maximum stress of seal ring and setting force with different seal ring thickness.

Through a comprehensive simulation analysis of metal sealing ring thickness variants at 8.4 mm, 9.4 mm, and 10.4 mm, we derived critical relationship curves portraying the interplay between sliding position shift, setting force, and the ensuing maximum stress exerted on the metal sealing ring during the ball seat setting process, as elucidated in Fig 8. The analysis reveals a direct correlation between sealing ring thickness and operational dynamics; notably, an increase in thickness limits the sliding body’s movement necessary for achieving sealing contact against the casing wall, thereby necessitating an elevation in the setting force applied by the soluble ball seat.

Specifically, for sealing rings with thicknesses of 8.4 mm and 10.4 mm, maximum stress remains below the 80 MPa threshold, indicating structural safety. In contrast, at 9.4 mm thickness, stress increases to 146.8 MPa - exceeding the material’s allowable stress limit and indicating potential safety risks to structural integrity, particularly in the sealing ring’s groove region.

Table 3 further shows how sealing effectiveness varies with sealing ring thickness. At 9.4 mm and 10.4 mm thicknesses, sealing performance—measured by contact pressure against both casing wall and sliding body interface—significantly improves compared to 8.4 mm configurations. Interestingly, sealing performance shows no substantial difference between 9.4 mm and 10.4 mm thicknesses. Notably, while peak contact pressure at 10.4 mm exceeds the 9.4 mm value, the average contact pressure decreases, indicating that sealing performance improvements at greater thicknesses do not increase linearly. Consequently, overall performance assessment demonstrates superior sealing outcomes at both 9.4 mm and 10.4 mm thicknesses, with nuanced preference depending on specific application requirements for pressure resilience and operational reliability.

**Table 3:** Sealing index of seal ring with different seal ring thickness.

$d_{1}$	$P_{max1}$ (Mpa)	$P_{mean1\;}$(MPA)	$\sigma_{1}$	$P_{max2}$ (Mpa)	$P_{mean2}$ (Mpa)	$\sigma_{2}$
8.4	27.78	11	119.561	26.7	10.98	89.042
9.4	54.45	27.9	191.978	37.03	20.07	75.875
10.4	56	27.24	86.205	49.35	16.76	85.423

#### 5.2.2. Groove angle (θ1) considerations.

When evaluating how different inclination angles (20°, 30°, and 40°) affect the groove of the sealing ring, consistent patterns emerge regarding tonnage reduction and associated maximum stress levels. Specifically, at 20°, 30°, and 40° inclination angles, the tonnage reduction values are 4.61t, 4.69t, and 4.78t respectively, while the corresponding maximum stresses measure 117.52MPa, 132.07MPa, and 126.67MPa. Notably, the 20° angle exhibits slightly lower maximum stress than the 30° and 40° configurations, indicating moderately improved stress distribution at this angle. This finding is significant given that the tonnage reduction remains relatively stable across all tested angles, suggesting minimal impact on the material’s compression characteristics.

**Fig 9:**
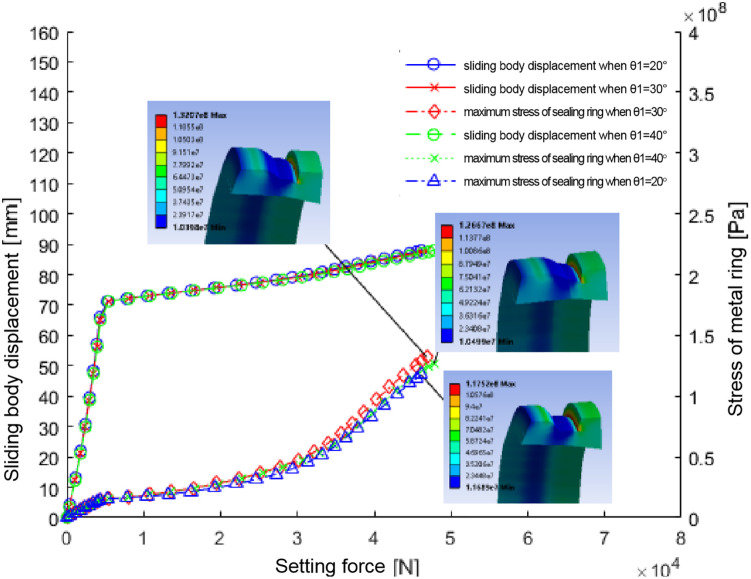
Diagram of relationship between sliding body displacement, maximum stress of seal ring and setting force with different upper side dip in the groove.

Moreover, the evaluation demonstrates an optimally balanced contact pressure distribution between the sealing ring and the sliding body, accompanied by significantly reduced stress concentrations, which are most pronounced at the 20° inclination angle. This balance manifests in comparatively higher average contact pressure at the interfaces between the sealing ring, casing wall, and sliding body. Consequently, these conditions correspond to lower reduction tonnage and reduced stress levels on the sealing ring. As illustrated in Fig 9 and detailed in Table 4, these results establish the 20° inclination angle (θ1 = 20°) as the recommended groove geometry for the sealing ring.

**Table 4:** Sealing index of seal ring with different upper side dip in the groove.

θ1(°)	P_max1_(Mpa)	P_mean1_(Mpa)	σ1	P_max2_(Mpa)	P_mean2_(Mpa)	σ2
20	53.58	26.36	198.207	35.98	19.73	81.364
30	52.14	25.76	191.978	37.50	17.91	75.875
40	52.23	25.44	191.137	37.26	18.81	69.86

This preference stems from the angle’s efficacy in enhancing sealing ring performance by ensuring adequate contact pressure distribution while concurrently mitigating stress concentrations—key factors that are instrumental in optimizing the structural integrity and operational longevity of the sealing mechanism.

#### 5.2.3. The impact of groove depth (D2) on the sealing performance.

The study on the effect of groove depth (D2) variations—spanning 1.8 mm, 2.8 mm, and 3.8 mm—on the metal seal ring reveals a direct correlation between the depth and both the reduction tonnage and the maximal stress experienced by the seal ring. Specifically, measurements showed reducing tonnages of 4.99t, 4.91t, and 4.83t and maximal stresses of 98.3Mpa, 84.2Mpa, and 79.3Mpa for the three depths respectively. This trend underscores that an augmentation in the groove depth translates to a decrement in both the reduction tonnage required and the principal stress acting on the seal ring. Furthermore, while an increase in the groove depth results in a lower contact pressure between the seal ring and the casing wall, it also brings about a more uniform pressure distribution across the sealing interface, as indicated by the decrease in variance; this suggests an enhanced sealing efficiency and diminished stress concentration within the seal ring.

**Fig 10:**
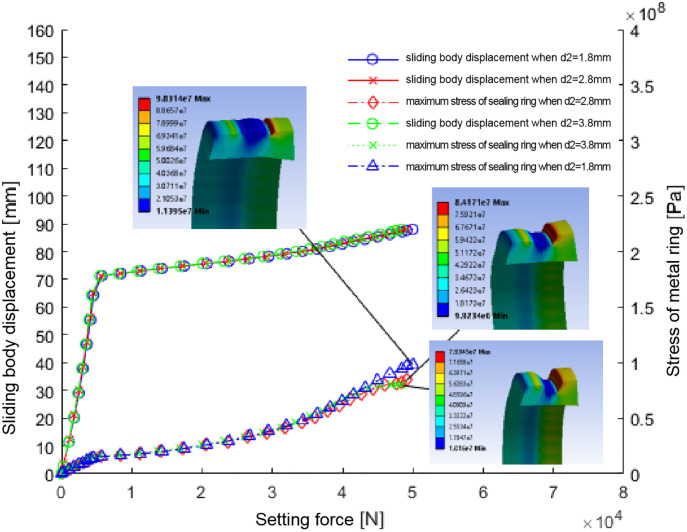
Diagram of relationship between sliding body displacement, maximum stress of seal ring, and setting force across different groove depths.

As illustrated in Fig 10 and quantified in Table 5, these parameter interactions highlight the complex relationship between groove depth, sliding body displacement, maximum seal ring stress, and operating forces. This interdependence is critical for optimizing seal performance across diverse pressure conditions and operational scenarios.

**Table 5:** Sealing Indices for Seal Rings at Varied Groove Depths.

d2(mm)	P_max1_(Mpa)	P_mean1_(Mpa)	σ1	P_max2_(Mpa)	P_mean2_(Mpa)	σ2
1.8	75.92	30.25	291.63	34.9	20.83	36.171
2.8	55.79	28.2	232.315	35.34	20.38	49.807
3.8	51.85	26.91	193.369	36.14	20.02	76.792

Considering the observed stress concentrations within the seal ring’s groove region, operational safety requirements for the soluble ball seat favor groove depths of 3.8 mm or 2.8 mm. These depths reduce necessary compression tonnage while improving stress distribution, thereby extending seal longevity and enhancing reliability in high-pressure applications.

The analysis confirms groove depth’s central role in designing and optimizing metal seal rings, particularly for systems experiencing variable pressures and mechanical stresses. This design parameter is essential for ensuring superior sealing integrity and operational safety.

#### 5.2.4. The influence of groove lower roll angle (θ2) on sealing performance.

**Fig 11:**
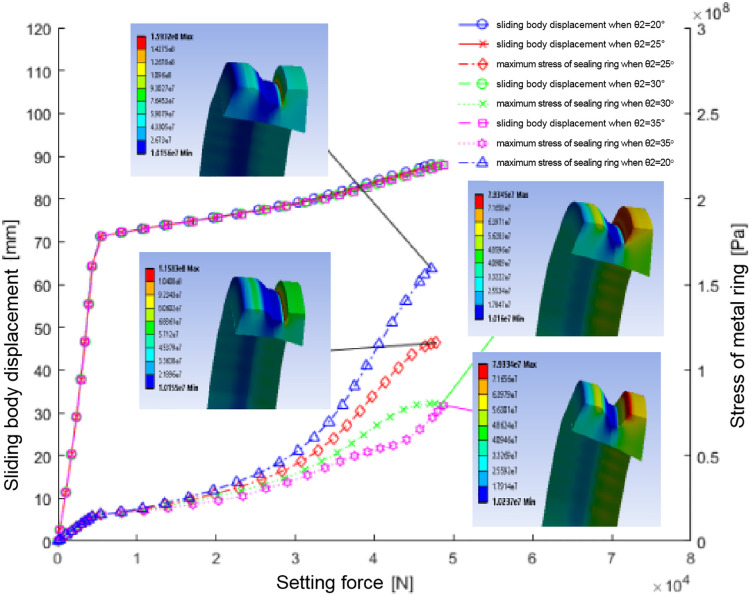
Portrays the relationship between sliding body displacement, maximum stress of the sealing ring, and setting force relative to differing lower groove inclination angles.

Analysis of the lower roll angle (θ2) influence on sealing ring groove performance, as demonstrated in Fig 11 and quantified in Table 6, shows that angles of 20°, 25°, 30°, and 35° produce maximum stresses of 159.32MPa, 115.83MPa, 79.35MPa, and 79.33MPa respectively. This trend confirms that increasing θ2 significantly reduces maximum groove stress, thereby improving the structural integrity and operational safety of the sealing ring.

**Table 6:** Seal Ring Sealing Indices for Various Lower Roll Angles (θ2).

θ2(°)	P_max1_(Mpa)	P_mean1_(Mpa)	σ1	P_max2_(Mpa)	P_mean2_(Mpa)	σ2
20	52.87	24.76	201.144	36.98	19.97	74.035
25	52.72	25.77	197.568	36.96	20.07	78.262
30	51.85	26.92	192.176	36.14	20.02	65.92
35	64.37	28.42	275.27	35.77	20.08	64.585

Analysis investigations reveal that a lower roll angle (θ2) of 35° enables the metal ring to attain maximum average contact pressures of 64.37 MPa with the casing wall and 35.77 MPa with the sliding body, representing peak values among all tested configurations. The observed positive correlation between increased groove inclination angle and enhanced interfacial contact pressure demonstrates a direct improvement in sealing performance. Mechanical analysis confirms that this 35° geometry simultaneously achieves two critical design objectives: significant reduction in stress concentration factors at the groove region. These synergistic effects collectively enhance both the structural integrity and operational reliability of the sealing system. Comprehensive evaluation of analysis results conclusively identifies the 35° lower roll angle as the optimal configuration, providing higher mean contact pressure compared to conventional designs while ensuring more uniform pressure distribution, thereby establishing this geometry as the technically superior solution for high-performance sealing applications requiring both mechanical durability and fluid containment reliability.

#### 5.2.5. Effect of contact surface inclination between sealing ring and sliding body (θ3) on sealing performance.

**Fig 12:**
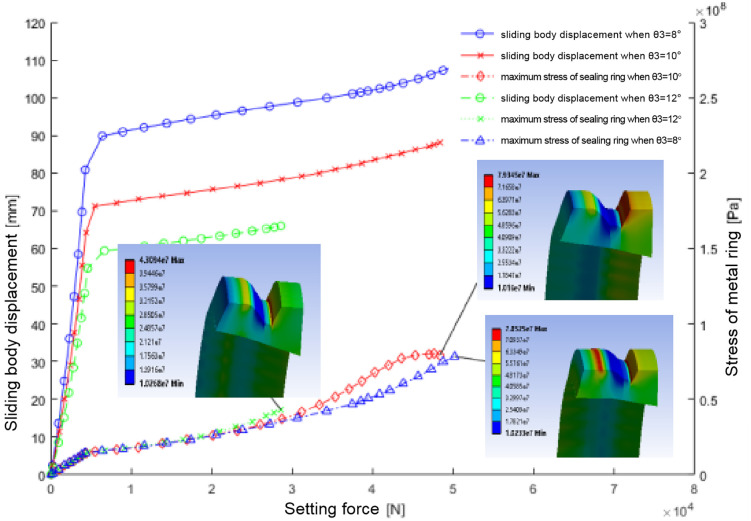
Demonstrates the relation between sliding body displacement, maximum stress on the sealing ring, and setting force across different contact angles between the sealing ring and the sliding body.

Analysis presented in Fig 12 and quantified in Table 7 reveals the critical relationship between the contact surface inclination angle (θ3) and sealing performance. Data demonstrate that increasing θ3 reduces both the crown thickness of the sealing ring and its required seating pressure. Specifically, at 12° inclination, the sealing ring develops significantly lower maximum stress compared to configurations with 8° and 10° angles.

**Table 7:** Sealing Ring Sealing Indices for Various Contact Angles (θ3).

θ3(°)	P_max1_(Mpa)	P_mean1_(Mpa)	σ1	P_max2_(Mpa)	P_mean2_(Mpa)	σ2
8	67.41	30.76	184.231	33.19	21.26	45.21
10	51.85	26.92	192.176	36.14	20.02	65.92
12	26.16	13.02	32.149	18.66	10.3	8.027

The analysis demonstrates that increasing θ3 progressively thins the crown region of the sealing ring while simultaneously reducing average contact pressure across the sealing ring-sliding body-casing wall interfaces. Although the 12° inclination minimizes seal ring stress, this configuration concurrently causes substantial contact pressure reduction, resulting in inadequate interfacial contact between these components and consequently failing to meet required seal quality under high-pressure conditions.

The findings establish that contact angles of 8° or 10° between the sealing ring and sliding body provide the optimal configuration. These angles balance maximum stress reduction in the sealing ring with adequate interfacial contact pressure for reliable sealing performance, ensuring the sealing mechanism meets operational standards while preventing unacceptable stress concentrations.

#### 5.2.6. Impact of the contact surface inclination between sealing ring and casing wall (θ4) on sealing performance.

**Fig 13:**
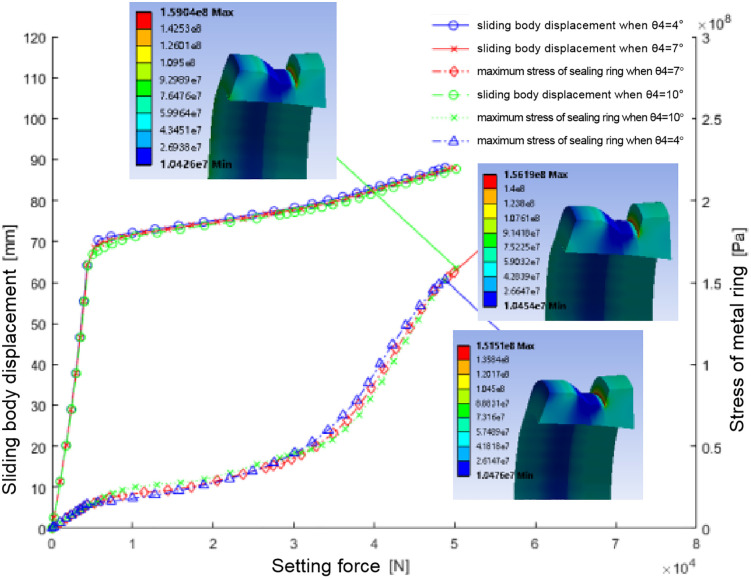
Plots the relationship between sliding body displacement, maximum stress on the seal ring, and setting force across different contact angles between the sealing ring and the casing wall.

The research detailed in Fig 13 and summarized in Table 8 focuses on how the contact surface inclination (θ4) between the sealing ring and the casing wall influences the sealing efficacy. Examining the inclinations at 4°, 7°, and 10°, it’s observed that the reducing tonnage required by the sealing ring exhibits a gradual increase, recorded at 4.88t, 4.98t, and 5.02t, respectively. Concurrently, the maximum stress experienced by the seal ring escalates slightly, noted as 151.51Mpa, 156.19Mpa, and 161.47Mpa for each inclination respectively.

**Table 8:** Seal Ring Sealing Indices for Various Contact Angles (θ4).

*θ*4(°)	P_max1_(Mpa)	P_mean1_(Mpa)	σ1	P_max2_(Mpa)	P_mean2_(Mpa)	σ2
4	52.25	30.47	149.870	36.45	22.24	79.482
7	53.19	31.49	152.158	45.85	23.19	96.051
10	53.46	31.56	139.717	67.16	24.06	142.74

The results demonstrate that greater inclination angles between the sealing ring and casing wall significantly increase contact pressure at the sealing ring-sliding body interface. Specifically, at 10° inclination, contact pressure between the sealing ring and casing wall exceeds levels recorded at 4°. Furthermore, this configuration reduces contact pressure variance, yielding substantially more uniform pressure distribution across the contact interface.

In conclusion, the optimal inclination angle between the sealing ring and casing wall is established at 10°. This configuration maximizes sealing efficiency through enhanced contact pressure and improved uniformity, while accepting a marginal increase in reduction tonnage and maximum stress—a technically acceptable trade-off given the overall performance gains. Crucially, this inclination ensures stable, uniform sealing contact that satisfies operational sealing standards while effectively managing stress concentrations.

#### 5.2.7. Orthogonal optimization experiment for sealing ring structural parameters.

An orthogonal optimization experiment was conducted to identify the best combination of structural parameters for the sealing ring following a preliminary single-variable analysis. This analysis revealed that certain dimensions and angles—specifically, a seal ring thickness of d1 = 9.4 mm or 10.4 mm; a groove inclination (θ1) of 20°; groove depths (d2) of 3.8 mm or 2.8 mm; a groove lower inclination (θ2) of 35°; contact surface inclinations between the sealing ring and sliding body (θ3) of 8° or 10°; and an inclination between the sealing ring and casing wall (θ4) of 10°—result in superior sealing effects. To refine these parameters, an L4 (2^3^) orthogonal array was utilized, summarizing the test into a three-factor, two-level analysis as delineated in Table 9.

**Table 9:** Seal Ring Structural Parameter Combinations.

Combination	Structural parameters of sealing ring		
	d1(mm)	d2(mm)	θ3(°)
1	9.4	2.8	8
2	9.4	3.8	10
3	10.4	2.8	10
4	10.4	3.8	8

**Fig 14:**
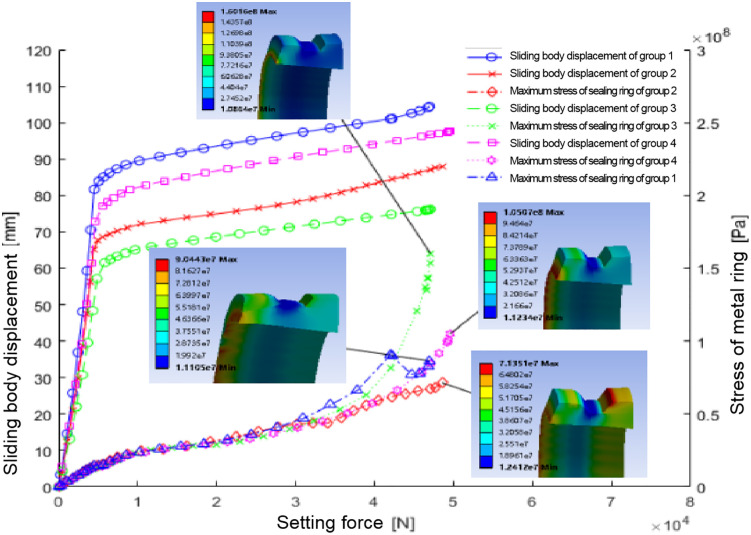
Presents the outcomes of this analysis, illustrating the relationship between sliding body displacement, seal ring maximum stress, and setting force across different structural parameter combinations.

Through the simulation analysis, the relationship curve between the displacement of the sliding body and the setting force during the setting process of each combined ball socket, and the relationship curve between the maximum stress of the main body of the metal seal ring and the setting force are obtained, as shown in Fig 14.

The data emphasize the significant impact of seal ring thickness and contact surface inclination on the maximum stress exerted on the seal ring. A thickness of 9.4 mm showed lower maximum stress compared to 10.4 mm. Moreover, a 10° contact surface inclination yielded less stress than an 8° inclination. Contact pressures for each parameter combination are given in Table 10, revealing higher contact pressures for seal ring combinations 1 and 2 against the casing wall compared to combinations 3 and 4. Thus, at a thickness of 9.4 mm, the seal ring is more inclined to compress and effectively contact the casing wall.

**Table 10:** Sealing Indices across Different Seal Ring Parameter Combinations.

PC	P_max1_(Mpa)	P_mean1_(Mpa)	σ1	P_max2_(Mpa)	P_mean2_(Mpa)	σ2
1	55.32	27.91	90.03	75.65	21.88	270.55
2	52.62	28.16	126.87	52.73	19.59	111.00
3	42.25	20.26	63.64	181.02	22.07	839.51
4	49.61	23.88	82.77	72.51	18.59	221.37

Through orthogonal experimental design, the structural parameters of the seal ring are optimized: the thickness of the seal ring d1 = 9.4 mm, and the inclination angle of the seal ring groove θ1 = 20°, sealing ring groove depth d2 = 3.8 mm, sealing ring groove lower inclination θ2 = 35°, inclination of contact surface between sealing ring and sliding body θ3 = 10°, inclination of contact surface between sealing ring and casing wall θ4 = 10°.

## 6. Experimental analysis of setting performance for metal sealing rings

This analysis evaluates the setting performance of metal sealing rings made from Material 1 and Material 2. We utilize a standard electro-hydraulic servo universal testing machine to investigate the setting safety of these rings under simulated field forces Table 11.

**Table 11:** The main technical specifications hydraulic universal testing machine.

Maximum Test Force (kN)	Test Force Accuracy(%)	Piston Stroke (mm)	Displacement Measurement Accuracy (%)	Loading Speed (mm/min)
300	±0.5	250	±0.5	0.01 ~ 100

### 6.1. Test apparatus and materials

**Fig 15:**
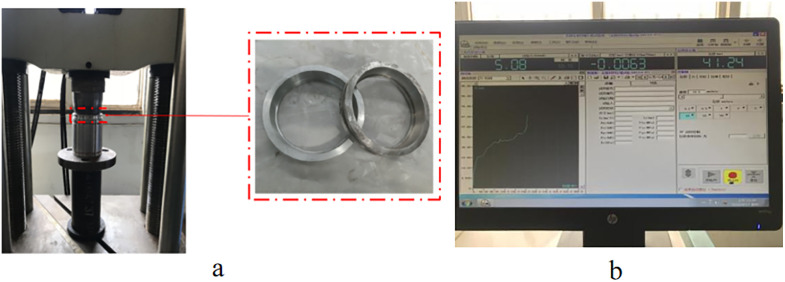
Test equipment. **(a)** Testing Machine and Metal Sealing Ring. **(b)** Data Processing Equipment.

The testing assembly, illustrated in Fig 15, encompasses a universal testing machine (model 1908112, HDT106A), a set of metal rings designated as 1 and 2, alongside data processing apparatus.

### 6.2. Procedures and methodology

(1) Fabrication of metal rings

The proportions for Material 1 and Material 2 are meticulously measured before undergoing smelting and sintering. Following this, vibration and rapid prototyping processes ensure uniform composition and refined grain structure. Optimal quenching and artificial aging treatments yield the final metallic rings.

(2) Setup and calibration of testing and tooling equipment;
(3) Configuration of the data acquisition system and mechanical test parameters;
(4) Load application: The applied force on the testing machine ranges from 0–6 tons, with accompanying sliding body displacement from 0–90 mm. Observations are made for the incidence of ring fissures;
(5) Collection and transmission of test data;
(6) Data evaluation and interpretation.

### 6.3. Results Interpretation

**Fig 16:**
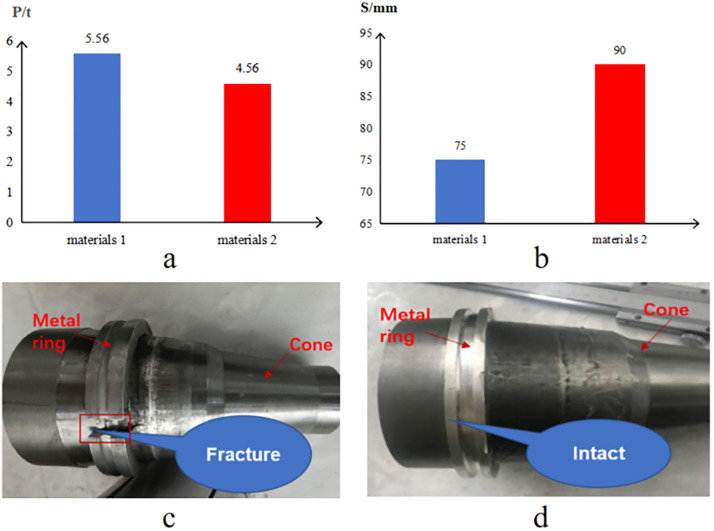
Comparative assessment of both materials. (a) Setting force. (b) Displacement. (c) Material 1 test (failure). (d) Material 2 test (successful).

Utilizing the electro-hydraulic servo universal tester for setting force application, we acquire and interpret setting force and displacement data for the metal rings. Fig 16 displays histograms that depict the outcomes of Material 1’s and Material 2’s test.

From the comparative tests on ring narrowing for both materials, we deduce the following:

(1) The maximum tonnage required for Material 1’s ring reduction is 5.56 tons, surpassing the its 5-ton threshold. The exerted force exceeds maximum setting force requirements. At this tonnage, a peak sliding displacement of the cone at 75 mm causes the ring to fracture, indicating subpar setting security and test failure.
(2) Material 2’s ring endures a maximum reduction force of 4.56 tons, within the 5-ton span. The peak sliding cone displacement reaches 90 mm without any fissuring, implying reliable setting safety and test success.
(3) Post-reduction, Material 2’s average outer diameter measures 125.36 mm; its seal ring remains unscathed, which is less than the inner diameter expansion of 1.1 mm for a standard 5 ½″ casing, permitting an advantageous metal seal fit.

## 7. Discussion

(1) Based on the comparative analysis of diameter-adjustment tonnage, maximum contact stress, and average contact stress on the interface between the two metallic ring materials, Material 2 demonstrates superior elongation characteristics. When employed as the metallic ring material, Material 2 exhibits enhanced deformability and requires lower diameter-adjustment tonnage, while structural optimization can be implemented to further increase contact pressure. Simulation results reveal that the maximum stress in Material 1’s metallic ring occurs at the sliding body compression zone, whereas Material 2 exhibits significantly reduced stress in this critical region. However, Material 2’s ring develops peak stress concentration at the right-angled groove in its central section, which can be effectively mitigated through structural modifications to simultaneously improve both safety performance and sealing effectiveness. Consequently, Material 2 emerges as the primary preferred material for metallic ring applications, with its performance advantages being further amplifiable through targeted geometrical optimizations of stress-critical features.
(2) A study on the influencing factors of sealing performance for novel structural metal rings was conducted, analyzing the influence laws of structural parameters on the sealing performance of metal rings. The analytical results demonstrate that when the metal ring thickness d1 is 9.4 mm or 10.4 mm, the contact stress between the metal ring and both the casing wall and sliding body increases, resulting in more reliable sealing; when the upper groove angle θ1 of the metal ring is 20°, the stress on the metal ring is reduced, ensuring better structural safety; when the groove depth d2 is 3.8 mm or 2.8 mm, the stress on the metal ring decreases, enhancing structural safety; as the lower groove angle θ2 increases, the maximum equivalent stress on the metal ring decreases while the average contact stress between the metal ring and the casing wall increases, making θ2 = 35° the preferred choice; when the contact surface angle between the metal ring and the sliding body θ3 is 8° or 10°, the contact stress between the metal ring and both the casing wall and sliding body increases; With an increase in the contact surface angle θ4 between the metal ring and the casing wall, the maximum equivalent stress on the metal ring slightly rises while the average contact stress between the metal ring and the casing wall increases. Therefore, when θ4 = 10°, this structure significantly enhances the structural integrity and sealing performance of the metal ring.
(3) The structural optimization of the metal ring was conducted based on the orthogonal experimental method, and the optimal parameter combination was determined as follows: metal ring thickness d1 = 9.4 mm, upper groove angle θ1 = 20°, groove depth d2 = 3.8 mm, lower groove angle θ2 = 35°, contact surface angle between metal ring and sliding body θ3 = 10°, and contact surface angle between metal ring and casing wall θ4 = 10°. Under this optimized parameter combination, the maximum stress of the metal ring was significantly reduced compared to the pre-optimization value. The maximum contact stress between the metal ring and casing wall was 52.62 MPa, while that between the metal ring and sliding body was 52.73 MPa, ensuring reliable sealing during the fracturing process.

## 8. Conclusions

(1) This study presents design improvements for soluble ball seat metal seal rings optimized for 124.26 mm inner diameter casings. Structural refinements—specifically widening the sliding body interface while adjusting upper and lower groove inclination angles—achieve uniform casing wall contact pressure distribution with concurrently reduced maximum seal ring stress. Additionally, a comprehensive evaluation framework integrating structural strength criteria and contact stress analysis was developed to assess sealing ring performance, establishing a robust methodology for future optimization.
(2) The optimized seal ring design—featuring 9.4 mm thickness, 20° upper groove angle, 3.8 mm groove depth, 35° lower groove angle, and consistent 10° contact surface angles—achieves uniform pressure distribution while maintaining stress below material limits under 50MPa pressure differential. This configuration demonstrates superior sealing performance while exhibiting robust structural integrity, reliably withstanding 50MPa operational pressure after installation.
(3) The modified Al-Mg/Ga alloy (Material-2) outperforms conventional Al-Mg alloy (Material-1), demonstrating superior displacement characteristics and reduced installation force requirements as confirmed by finite element analysis and experimental validation. Consequently, Material-2 sealing rings deliver enhanced operational safety, ensuring reliable seating integrity under service conditions. Collectively, these findings represent significant advancements in downhole sealing technology, offering improved functional reliability for future applications.
